# Artificial intelligence‐based diagnosis of non‐displaced femoral neck fractures shows excellent sensitivity and specificity and reduces the need for computed tomography scans in emergency rooms

**DOI:** 10.1002/jeo2.70781

**Published:** 2026-05-26

**Authors:** Mikhail Salzmann, Michelle Radharani Diallo, Robert Prill, Roland Becker, Andreas G. Schreyer, Marko Ostojic, Nikolai Ramadanov

**Affiliations:** ^1^ Centre of Orthopaedics, Traumatology and Plastic Surgery Brandenburg Medical School, University Hospital Brandenburg an der Havel Brandenburg an der Havel Germany; ^2^ Faculty of Health Science Brandenburg Brandenburg Medical School Theodor Fontane Brandenburg an der Havel Germany; ^3^ Institute for Diagnostic and Interventional Radiology, Brandenburg Medical School Theodor Fontane Brandenburg an der Havel Germany; ^4^ Sports Traumatology Division, Traumatology Department “Draskoviceva” University Hospital “Sisters of Mercy” Zagreb Croatia

**Keywords:** artificial intelligence, computed tomography, deep learning, diagnostic accuracy, femoral neck fracture

## Abstract

**Purpose:**

The purpose of this study was to evaluate the diagnostic performance of an artificial intelligence (AI)‐based system for the detection of non‐displaced femoral neck fractures (FNFs) on plain pelvic radiographs and to assess its potential impact on confirmatory computed tomography (CT) utilization.

**Methods:**

In this retrospective case‐control single‐centre study, 394 anteroposterior (AP) pelvic radiographs were analysed, including 197 patients with clinically confirmed non‐displaced FNFs (Garden I–II) and 197 control radiographs without fracture. Radiological reports and AI outputs were categorized as positive, negative or doubtful. Doubtful findings were analysed separately. Diagnostic accuracy metrics were calculated, and paired comparisons between radiologists and AI were performed using McNemar's test. A secondary analysis evaluated the theoretical potential of AI to reduce confirmatory CT imaging.

**Results:**

The AI software correctly identified 189 of 197 fractures (95.9%) with a false‐negative rate of 1.0%, compared with 160 correct detections (81.2%) and a false‐negative rate of 7.6% by radiologists. McNemar's test demonstrated significantly fewer missed fractures by the AI software (*p* < 0.001). Using a combined radiologist–AI decision rule, only two fractures were missed, resulting in a combined miss rate of 1.0%. Among fracture patients undergoing CT, the AI provided a definitive positive result in 94.0% of cases, suggesting a substantial proportion of CT examinations might have served confirmatory purposes.

**Conclusion:**

Combined radiologist and AI‐assisted assessment in the evaluation of non‐displaced FNFs on plain AP pelvic radiographs improves the likelihood of detection and accurate diagnosis. The combined assessment yields the lowest miss rate and may reduce the need for confirmatory CT imaging without compromising patient safety, particularly in emergency settings and during off‐hours.

**Level of Evidence:**

Level III.

**Abbreviations**
AI: artificial intelligence
AP: anteroposterior
CI: confidence interval
CT: computed tomography
DHS: dynamic hip screw
FN: false negatives
FNF: femoral neck fracture
FP: false positives
HA: hip arthroplasty
LR+: positive likelihood ratio
LR−: negative likelihood ratio
MRI: magnetic resonance imaging
NPV: negative predictive value
PFN: proximal femoral nail
PPV: positive predictive value
ROC: receiver operating characteristic
SD: standard deviation
STARD: Standards for Reporting Diagnostic accuracy in studies
TN: true negatives
TP: true positives

## INTRODUCTION

Femoral neck fractures (FNFs) are among the most common fractures in the elderly population [25]. Their incidence is expected to rise further as the global population continues to age [11, 14]. Several studies have demonstrated that delayed surgery is associated with higher rates of adverse events such as prosthetic joint infections and increased mortality [21, 37]. A meta‐analysis has shown that early surgical intervention is associated with a significantly lower risk of death [35]. Therefore, fast and accurate diagnosis is critical.

Non‐displaced FNFs remain diagnostically challenging, particularly because these patients frequently present to emergency departments during night shifts [12], and misdiagnosis rates of up to 7%–14% have been reported [6, 30]. Although computed tomography (CT) or magnetic resonance imaging (MRI) can increase diagnostic accuracy, these modalities are not always available, require more resources and may delay treatment [29].

Artificial intelligence (AI) applications are rapidly emerging in medical imaging, with several studies reporting high diagnostic accuracy [17, 31]. Approaches range from self‐developed algorithms [36] to commercially available vendor‐provided solutions [13, 27]. AI‐based tools have also been shown to improve the diagnostic performance of radiology residents in detecting hip fractures [32]. A recent multilevel meta‐analysis demonstrated excellent overall accuracy of AI‐guided detection of FNFs; however, most included studies did not differentiate between displaced and non‐displaced fractures [28]. As a result, the accuracy of AI‐based detection specifically for non‐displaced FNFs—despite being the more diagnostically challenging subtype—remains underrepresented in the current literature.

The aim of this study was to investigate the diagnostic performance of an AI‐based tool for detecting non‐displaced FNFs (Garden Types I and II). We hypothesized that the integration of AI into the diagnostic workflow would improve diagnostic accuracy and reduce the need for additional CT imaging in uncertain cases.

## METHODS

### Study design and ethical approval

This retrospective single‐centre diagnostic accuracy study was designed as a case‐control investigation comparing radiologist reports and an AI‐based system for the detection of non‐displaced FNFs. The reference standard was the final clinical diagnosis at hospital discharge. The study was conducted in accordance with the principles of the Declaration of Helsinki. Ethical approval was obtained from the institutional review board (reference number 231072024‐BO‐E‐RETRO). All data were analysed in anonymised form. Informed consent was not required. Reporting followed the Standards for Reporting Diagnostic accuracy in studies (STARD) 2015 recommendations; items not applicable to the retrospective design were addressed where possible [5].

### Participant selection and eligibility

The hospital information system was screened for all patients who consulted the emergency department between January 2014 and January 2025 and were discharged with a diagnosis of either a FNF or pelvic contusion. All patients who underwent an anteroposterior (AP) pelvic radiography during this encounter were identified. Two orthopaedic surgeons (M. S. and M. R. D.) then manually viewed all screening results. Patients were included if they had (I) an AP pelvic radiography, and (II) either a non‐displaced FNF (Garden Type I or II) or no fracture (pelvic contusion). When a CT scan of the pelvis or hip was performed on the same day, this was documented separately. Patients were excluded if they had (I) a displaced FNF (Garden III–IV), (II) fractures of the pelvic ring or other lower limb regions (e.g., fractures of the greater trochanter), (III) periprosthetic or periosteosynthetic fractures or (IV) underwent CT only without plain radiography. The presence of implants on the contralateral side (total hip arthroplasty or intramedullary nails) was not defined as an exclusion criterion but was recorded during data extraction. As this was a retrospective study including all eligible fracture cases during the predefined study period, no a priori sample size calculation was performed. Control cases were randomly selected to achieve a balanced case‐control ratio.

### Data acquisition

A total of 197 radiographs from 197 patients with non‐displaced FNFs were included. To form an equally sized control group, 197 radiographs from 178 patients with pelvic contusions and no fracture on final diagnosis were randomly selected. In this control group, 197 radiographs originated from 178 patients. Moreover, 19 radiographs were obtained from patients who presented more than once during the study period due to separate traumatic events. Each radiograph was treated as an independent diagnostic test instance. Ground truth was defined as the final diagnosis upon hospital discharge, independent of the radiological report.

BoneView™ (Version 2.7.0.1; Gleamer), a commercially available diagnostic support tool based on AI, has been used for this study. It is a CE‐marked medical device designed for the detection of fractures, effusions, dislocations and focal bone lesions on DICOM images. The algorithm covers the analysis of the lower and upper limbs, pelvis, thoracolumbar spine and rib cage for patients aged 2 years and above. The algorithm uses a convolutional neural network built from ‘Detectron2’. To develop the algorithm, a dataset of 500,000 patient radiographs from 22 radiology departments between January 2011 and May 2023 was used. This dataset was then randomly divided into 80% for training, 5% for validation and 15% for internal testing. The training dataset included 20% of patients under the age of 18 years. The algorithm works with two operating points: ‘doubtful’ when the confidence score is between 50% and 90%, and ‘positive’ when the confidence score exceeds 90%. A confidence score between 0% and 50% will yield a negative result. For user clarity, the software highlights the region of interest by using a rectangular box on the radiographs, using a continuous line for positive results and a dotted line to indicate doubt.

### Radiological report and AI processing

All included radiographs have been reviewed for their final radiological report (M. S. and M. R. D.) and categorized into three subgroups: positive, negative and doubtful. BoneView™ was applied to the same radiographs, and the AI output was exported categorically in the same three subgroups. Doubtful findings were analysed as a separate category and were not forced into binary classification. It is important to note that CT scans were often performed based on clinical decision‐making by the trauma team, especially at night, and independently of the final radiological report. Radiographic interpretation was performed during routine clinical workflow by board‐certified radiologists and supervised radiology residents, reflecting real‐world emergency department practice. The AI system was applied using the manufacturer's default thresholds without recalibration or additional training on the present dataset.

### Outcome measures

The primary outcome measure was the diagnostic accuracy of the AI system compared with radiologists in detecting non‐displaced FNFs, as well as the theoretical combined performance of radiologists plus AI. The following values were calculated: true positives (TP), true negatives (TN), false positives (FP), false negatives (FN), sensitivity, specificity, positive predictive value (PPV), negative predictive value (NPV), accuracy and positive and negative likelihood ratios (LR+ and LR–). Doubtful cases were not classified as correct or incorrect but reported separately.

### Statistical analysis

All radiographs were analysed as paired observations (radiologist vs. AI) by an independent researcher (R. P.) who was not involved in the data extraction process. Presence of a FNF (reference standard) and index test outputs (radiologist report and AI output) were treated as categorical variables with three categories (positive/negative/doubtful). For diagnostic accuracy metrics, ‘doubtful’ results were reported separately and excluded from binary 2 × 2 contingency tables; therefore, accuracy estimates were calculated on determinately classified cases only. Proportions (sensitivity, specificity, predictive values, accuracy) are reported with 95% confidence intervals calculated using the Wilson score method. Paired comparisons between radiologists and AI regarding missed fractures and other selected paired categorical outcomes were performed using McNemar's test, focusing on discordant results. No missing data were present for the index tests or reference standard. Statistical analysis was conducted using Microsoft Excel and IBM SPSS. A secondary analysis evaluated the theoretical potential of AI to reduce unnecessary CT imaging. Receiver operating characteristic (ROC) curve analysis was not performed because the AI system was evaluated using predefined categorical operating points (negative/doubtful/positive) without application of continuous confidence scores or threshold optimization.

## RESULTS

### Patient characteristics and study cohort

**Figure 1:**
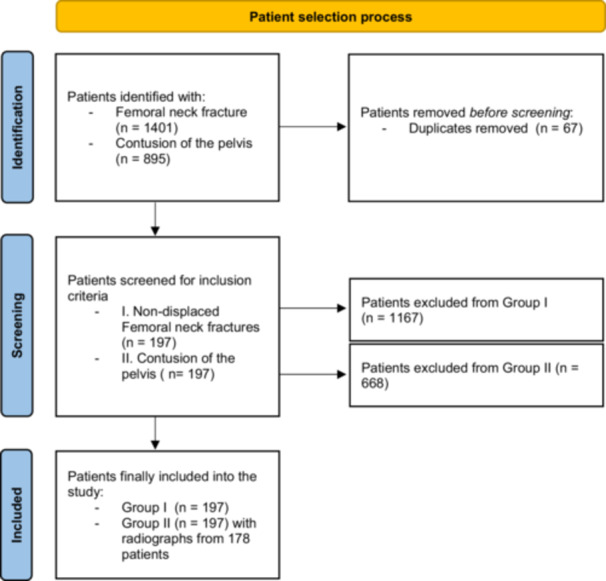
STARD flowchart highlighting the patient selection process. STARD, Standards for Reporting Diagnostic accuracy in studies.

1401 cases with FNF have been extracted from the hospital's database. After excluding duplicates, patients who had a displaced FNF (Garden Types III and IV), or subsets who met the exclusion criteria, 197 patients have been included in the group with a FNF. In the pelvic contusion group, a total of 895 cases have been screened. After removing duplicates and cases that did not match the inclusion criteria, patients from the control group were randomly selected. The selection process is shown in a STARD flowchart (Figure 1).

Finally, a total of 394 pelvic radiographs were included, comprising 197 patients with clinically confirmed non‐displaced FNFs (Garden I–II) and 197 radiographs from 178 patients with a pelvic contusion without fracture. CT was performed in 116 fracture cases and in 62 control cases, independent of the radiological report. The reference standard was the final clinical diagnosis at hospital discharge. The cohort consisted predominantly of elderly patients with a mean age of 77.30 ± 12.13 years in the fracture group and 72.25 ± 20.25 years in the control group. Baseline characteristics of the final study cohort are summarized in Table 1.

**Table 1:** Characteristics of the final study cohort.

Variable	Fracture group	Control group	*p* value
Age, mean ± SD (years)	77.30 ± 12.13	72.25 ± 20.25	0.003
Sex (m/f)	65/132	79/99	0.026
m:f ratio (males per female)	0.49	0.80	NA
Radiographs (unique patients)	197 (197 patients)	197 (178 patients)	NA
CT same day (*n*)	116	62	<0.001
Metalgear contralateral	21 (2 PFN, 2 DHS, 17 HA)	18 (4 PFN, 14 HA)	0.736

*Note*: Continuous variables are presented as mean ± SD and were compared using Welch's *t* test. Categorical variables were compared using Fisher's exact test. *p* Values are provided for descriptive comparison between groups in this retrospective case‐control cohort.

Abbreviations: AI, artificial intelligence; CT, computed tomography; DHS, dynamic hip screw; f, female; HA, hip arthroplasty; m, male; PFN, proximal femoral nail; NA, not applicable; SD, standard deviation.

### Diagnostic performance on plain radiographs

#### Fracture group (*n* = 197)

The AI system correctly identified 189 fractures as positive (95.9%), classified six cases as doubtful (3.0%) and missed two fractures (1.0%).

**Figure 2:**
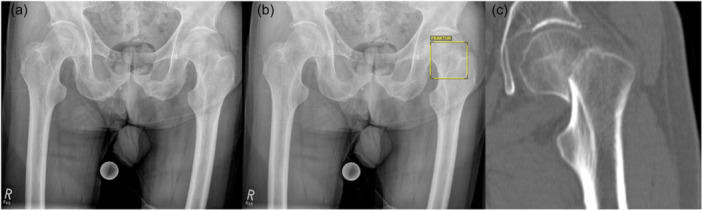
(a) Anteroposterior pelvic radiograph with example of a non‐displaced Femoral neck fracture initially not described in the radiological report. (b) Artificial Intelligence correctly identifying the fracture. (c) Confirmatory CT scan. CT, computed tomography.

Radiologists identified 160 fractures as positive (81.2%), classified 22 cases as doubtful (11.2%) and missed 15 fractures (7.6%). Thus, AI demonstrated a substantially higher rate of definitive fracture detection and a markedly lower false‐negative rate compared with radiologists. An example is presented in Figure 2.

#### Control group (*n* = 197)

In patients without fracture, the AI system classified 188 cases as negative (95.4%), produced one false‐positive result (0.5%) and classified eight cases as doubtful (4.1%).

Radiologists classified 191 cases as negative (97.0%), produced no false‐positive results and classified six cases as doubtful (3.0%). All outcome measures are presented in Table 2.

**Table 2:** Outcome measure synopsis with Wilson confidence intervals.

Outcome measure	Radiologist	AI	*p* value	Radiologist + AI
Sensitivity	91.4% (86.3–94.7)	99.0% (96.3–99.7)	<0.001	99.0% (96.4–99.7)
Specifity	100.0% (98.0–100.0)	99.5% (97.1–99.9)	1.0000	99.5% (97.2–99.9)
PPV	100.0% (97.7–100.0)	99.5% (97.1–99.9)	NA	99.5% (97.2–99.9)
NPV	92.7% (88.3–95.5)	98.9% (96.2–99.7)	NA	99.0% (96.3–99.7)
False‐negative rate	7.6% (4.8–11.9)	1.0% (0.3–3.7)	<0.001	1.0% (0.3–3.7)
Accuracy (overall)	95.9% (93.3–97.5)	99.2% (97.7–99.7)	NA	99.2% (97.8–99.7)
LR+	NA^a^	187.0 (26.5–1320)	NA	195.0 (27.6–1378)
LR−	0.086 (0.05–0.15)	0.010 (0.003–0.037)	NA	0.010 (0.003–0.037)
Doubt rate—fracture	11.2% (7.5–16.3)	3.0% (1.4–6.5)	0.004	0% (0.0–0.19)
Doubt rate—control	3.0% (1.4–6.5)	4.1% (2.1–7.9)	0.77	0.5% (0.1–2.8)
Doubts (total)—AI/Radiologist	7.1% (5.0–10.1)	3.6% (2.2–6.0)	0.038	0.5% (0.1–1.8)
Clear diagnosis rate (overall)	92.9% (89.9–95.0)	96.4% (93.9–97.9)	0.038	99.5% (98.2–99.9)

*Note*: Comparative *p* values between radiologist and AI assessments were calculated using the exact McNemar test for paired categorical outcomes. *p* values are shown only for clinically relevant and statistically meaningful paired comparisons.

Abbreviations: AI, artificial intelligence; LR+, positive likelihood ratio; LR−, negative Likelihood ratio; NA, not applicable; NPV, negative predictive value; PPV, positive predictive value.

^a^ Not available for radiologists due to the absence of false‐positive findings.

#### Combined performance of radiologist and AI

**Figure 3:**
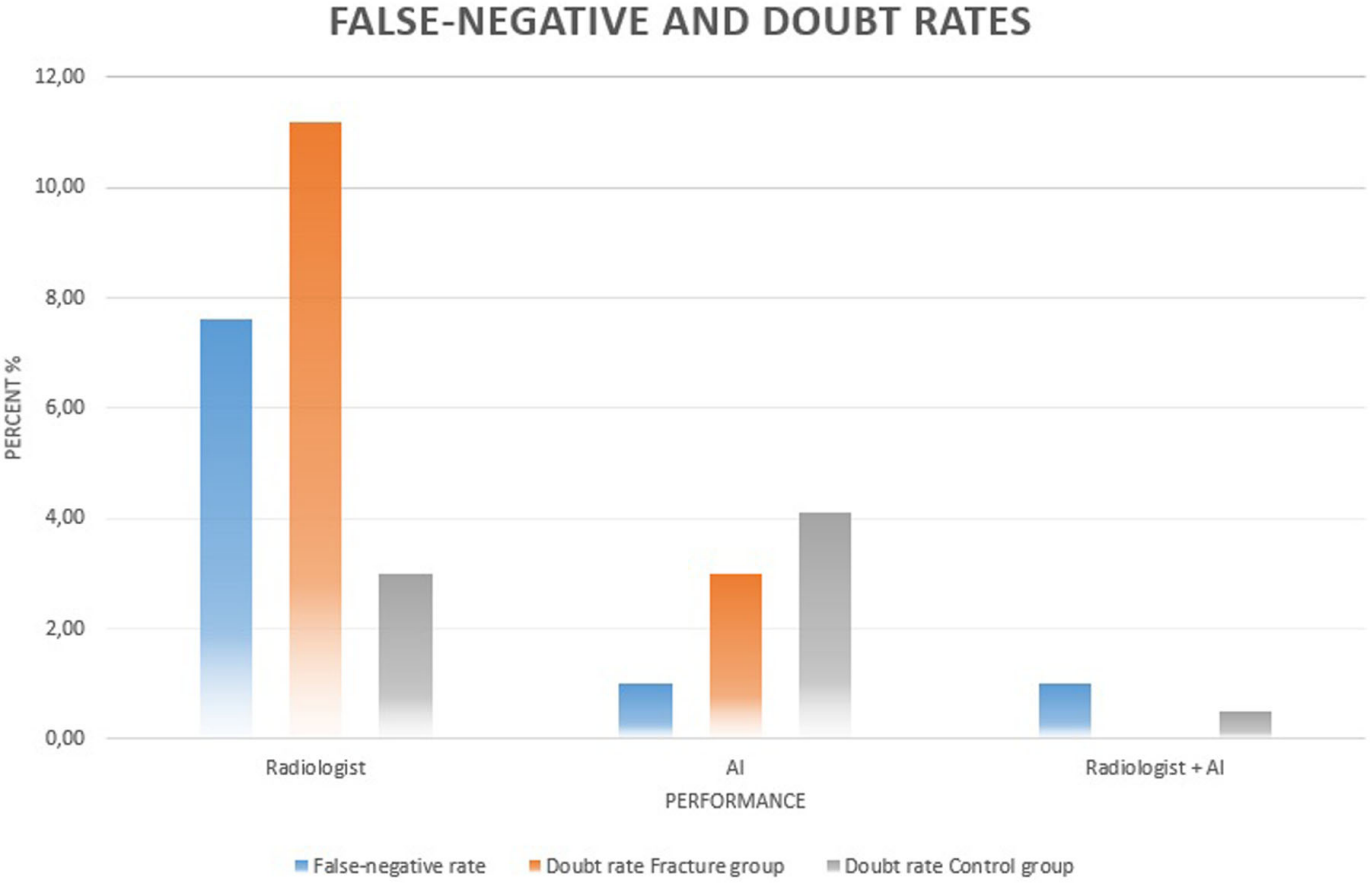
Bar chart illustrating false‐negative rates and doubt rates for the radiologist, the AI system and the combined assessment (radiologist + AI). Results are shown separately for the fracture group (*n* = 197) and the control group (*n* = 197). The combined strategy demonstrated the lowest false‐negative rate and the lowest rate of doubtful classifications in the fracture group. AI, artificial intelligence.

**Figure 4:**
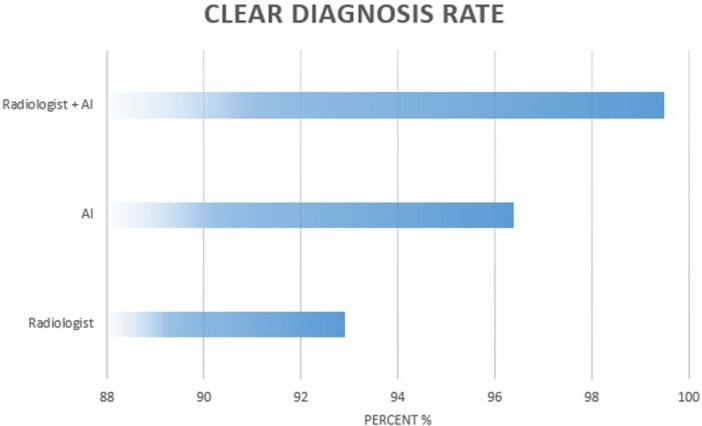
Bar chart showing the proportion of determinately classified radiographs (i.e., non‐doubtful assessments) for the radiologist, the AI system and the combined assessment. The combined strategy resulted in the highest rate of clear diagnostic classifications. AI, artificial intelligence

McNemar's test demonstrated that radiologists missed significantly more non‐displaced FNFs than the AI system (*p* < 0.001). Instead, using a theoretical combined decision rule (fracture detected if either the radiologist or the AI provided a definitive positive assessment), only two fractures were missed by both readers, resulting in a combined miss rate of 1.0%. Doubtful findings in fracture cases did not overlap between radiologists and AI, indicating complementary diagnostic behaviour. Therefore, the combined radiologist–AI assessment yielded the lowest miss rates among all evaluated approaches. Figure 3 illustrates the reduction in false‐negative and doubtful classifications, particularly when combining AI and radiologist assessment. Figure 4 demonstrates the resulting increase in clear diagnostic classifications.

### Potential of CT avoidance through AI‐based rule‐in

#### Fracture patients undergoing CT (*n* = 116)

Among fracture patients who underwent CT imaging, the AI system provided a positive result on the initial radiograph in 109 cases (94.0%), while six cases were classified as doubtful, and one fracture was missed. In comparison, radiologists provided a definitive positive assessment in only 85 of these cases (73.3%), with 20 doubtful and 11 false‐negative reports. Interpreting the data case‐by‐case, radiologists were non‐definitive in 31 cases (20 doubtful and 11 false‐negative). Only one fracture was occult on radiographs for both readers and was detectable on CT only. In 30 of those cases, however, the AI system provided a definitive positive result on the initial radiograph, indicating that these 30 CT examinations (25.8%) may primarily have served confirmatory purposes and might theoretically have been avoided.

#### Control patients undergoing CT (*n* = 62)

In control patients undergoing CT, the AI system produced no false‐positive results and classified five cases as doubtful. Consequently, only these five cases would likely have required confirmatory CT imaging based on AI classification alone to exclude a non‐displaced FNF, as the other 57 (91.9%) cases have been correctly classified by the AI as negative. Notably, doubtful assessments by the AI system and the radiologist overlapped in one case only, further underlining the complementary and synergistic potential of combined human–AI assessment.

## DISCUSSION

Combined radiologist and AI‐assisted analysis of pelvic radiographs demonstrated a substantially higher rate of definitive diagnosis of non‐displaced FNFs compared with either radiologist assessment or AI evaluation alone, thereby potentially reducing the need for confirmatory CT imaging without compromising patient safety. Importantly, this complementary diagnostic behaviour resulted in the lowest observed miss rate and highlights the value of AI as a decision‐support tool that augments, rather than replaces, radiologist expertise.

Delayed or missed diagnosis of non‐displaced FNFs remains a well‐documented clinical problem, particularly in emergency settings and during off‐hours [6, 23, 24]. Previous studies have reported miss rates ranging from 7% to 14%, especially for subtle or non‐displaced fractures [12, 30]. In line with these reports, radiologists in the present study demonstrated a non‐negligible rate of equivocal (11.2%) and false‐negative (7.6%) findings. In contrast, the AI system showed a markedly lower false‐negative rate (1%) and a substantially higher proportion of definitive positive classifications. These results are consistent with prior studies demonstrating high diagnostic accuracy of AI‐based fracture detection on plain radiographs [8, 22, 33]. By concentrating exclusively on Garden I and II fractures, the present study addresses a clinically relevant subgroup [3, 20].

Beyond stand‐alone diagnostic accuracy, our data highlight the complementary behaviour of human and AI assessment, which represents a key observation of this study. Equivocal and false‐negative cases showed minimal overlap between human readers and AI, resulting in a combined miss rate of only 1%. This finding supports previous evidence that AI performs best as a decision‐support tool rather than as an isolated measurement tool used without radiologist expertise [15, 32].

The combined radiologist–AI approach yielded the lowest miss rate among all evaluated strategies, supporting the concept that AI can enhance diagnostic robustness by compensating for human uncertainty or fatigue, particularly in borderline cases. Such synergy is of particular relevance in musculoskeletal trauma imaging, where subtle findings may be overlooked under time pressure [26].

In addition to diagnostic performance, a case‐by‐case analysis revealed that in a substantial proportion of fracture patients undergoing CT, radiologists were non‐definitive, whereas the AI system provided a clear positive diagnosis on the initial radiograph. In retrospect, these CT examinations might primarily have served confirmatory purposes. These findings align with previous literature where confirmatory CT or even MRI has been reported to be not uncommon in occult fractures or equivocal cases [9, 18, 23]. From a workflow perspective, this suggests that using an AI‐based rule‐in approach on plain radiographs, defined as a definitive positive AI classification, may reduce reliance on confirmatory CT imaging in selected cases, particularly when clinical suspicion is already high [16].

Conversely, in the entire control patient cohort, the AI system produced only one false‐positive result, indicating that CT avoidance through AI‐based rule‐in would not have significantly compromised patient safety.

Beyond diagnostic accuracy and complementary reader performance, the clinical relevance of these findings becomes particularly apparent when considering real‐world workflow conditions, healthcare resource allocation and the inherent limitations of current AI systems. Smaller hospitals and rural centres often rely on delayed teleradiology services, which may prolong diagnostic workflows and treatment decisions. In such settings, AI systems may be able to provide immediate, standardized decision support at the point of care, potentially bridging gaps in expertise and availability. This may not only reduce diagnostic delays but also decrease the need for patient transfers and additional imaging, thereby improving overall care efficiency [4, 7].

In addition to workflow implications, the potential health‐economic impact of AI‐assisted diagnostic pathways warrants consideration. Hip fractures impose a substantial economic burden on healthcare systems, with CT imaging adding further direct and indirect costs [2]. Although AI implementation involves initial and ongoing expenses, these may be relativized by reduced confirmatory CT use, improved diagnostic efficiency and decreased radiologist workload. By enabling reliable rule‐in diagnosis on plain radiographs, AI may help avoid confirmatory CT examinations, thereby lowering imaging‐related costs, radiation exposure, workflow delays and workload [10]. However, studies investigating the true benefits of AI in healthcare are limited and often focus on costs rather than health outcomes [34].

Lastly, the medico‐legal implications of AI‐assisted diagnostics remain an area of ongoing debate. Current evidence suggests that clinical responsibility remains with the interpreting physician, particularly when AI is used as a decision‐support system rather than an autonomous diagnostic tool [1, 19]. In cases of diagnostic uncertainty, decisions regarding further imaging or surgical treatment must therefore continue to rely on clinical judgement and institutional protocols. At present, AI cannot replace established diagnostic standards but may enhance diagnostic confidence and support more efficient resource utilization.

Despite these promising findings, several limitations related to AI‐assisted fracture detection must be acknowledged. The AI system showed occasional performance challenges in the presence of metallic implants and in distinguishing old from acute fractures. Although specificity remained high in the present cohort, such findings may affect specificity in broader clinical practice. In addition, CT examinations in emergency settings are not exclusively performed to confirm or exclude FNFs but may also serve to evaluate associated pelvic or acetabular injuries; therefore, the CT avoidance analysis should be interpreted as theoretical.

Furthermore, this retrospective single‐centre design may limit generalizability. Radiologist assessment was based on routine single‐reader reports, and no inter‐rater reliability analysis was performed.

Strengths of this study include (i) a clearly defined cohort with equal‐sized fracture and control groups, (ii) a strict focus on non‐displaced FNFs, (iii) the explicit separation of equivocal findings from binary outcome measures and (iv) the independent case‐by‐case verification of discordant findings as well as the structured assessment of CT utilization.

## CONCLUSION

AI‐assisted evaluation of pelvic radiographs was associated with improved detection of non‐displaced FNFs. The combination of AI and radiologist assessment shows the highest diagnostic performance and may reduce the need for confirmatory CT scans without compromising patient safety. These benefits are particularly relevant during night shifts and in hospitals with limited radiology resources. Future prospective studies are needed to validate these findings in real‐world clinical workflows and investigate possible benefits of AI more precisely. Therefore, AI should currently be regarded as a decision‐support tool rather than an autonomous diagnostic system, with the greatest benefit observed when AI and radiologist assessments are combined.

## AUTHOR CONTRIBUTIONS

All authors contributed to the study conception and design. Material preparation and data collection were performed by Mikhail Salzmann, Michelle Radharani Diallo and Nikolai Ramadanov. Analysis and statistics were performed by Mikhail Salzmann and Robert Prill. The first draft of the manuscript was written by Mikhail Salzmann and Nikolai Ramadanov. All authors commented on previous versions of the manuscript. All authors read and approved the final manuscript.

## FUNDING INFORMATION

The authors have no funding to report.

## CONFLICT OF INTEREST STATEMENT

The authors declare no conflicts of interest.

## ETHICS STATEMENT

Ethical approval was obtained from the institutional review board (reference number 231072024‐BO‐E‐RETRO).

## Data Availability

The dataset generated and/or analysed during the current study is available from the corresponding author upon reasonable request.
